# Disclosure of a Concealed Michelangelo-Inspired Depiction in a 16th-Century Painting

**DOI:** 10.3390/jimaging10080175

**Published:** 2024-07-23

**Authors:** Alice Dal Fovo, Margherita Morello, Anna Mazzinghi, Caterina Toso, Enrico Pampaloni, Raffaella Fontana

**Affiliations:** 1 National Research Council—National Institute of Optics (CNR-INO), Largo E. Fermi, 6, 50125 Florence, Italyraffaella.fontana@ino.cnr.it (R.F.); 2 OPD-Scuola di Alta Formazione e Studio, Via Alfani, 78, 50121 Florence, Italy; 3 Department of Physics and Astronomy, University of Florence, Via G. Sansone, 1, 50019 Sesto Fiorentino, Italy; 4 National Institute of Nuclear Physics (INFN), Florence Division, Via Bruno Rossi, 1, 50019 Sesto Fiorentino, Italy; 5 Opificio delle Pietre Dure, V.le Filippo Strozzi, 1, 50129 Florence, Italy

**Keywords:** painting, underpainting, underdrawing, Michelangelo, microprofilometry, X-ray fluorescence, reflectance spectroscopy, optical coherence tomography, spectral correlation mapping

## Abstract

Some paintings may have hidden depictions beneath the visible surface, which can provide valuable insights into the artist’s creative process and the genesis of the artwork. Studies have shown that these covered paintings can be revealed through image-based techniques and integrated data processing. This study analyzes an oil painting by Beceri from the mid-16th century depicting the Holy Family, owned by the Uffizi Galleries. During the analysis of the materials, we discovered evidence of pictorial layers beneath the visible scene. To uncover the hidden figuration, we applied a multimodal approach that included microprofilometry, reflectance imaging spectroscopy, macro X-ray fluorescence, and optical coherence tomography. We analyzed the brushstrokes of the hidden painting, visualized the underdrawing, located the painted areas beneath the outermost painting, and quantified the thicknesses of the pictorial layers. The pigments used for the underpainting were identified through cross-analysis of X-ray fluorescence and spectral correlation maps. The underlying pictorial subject, Leda and the Swan, appears to be inspired by a long-lost and replicated work by Michelangelo. This information places Beceri and his production in a more defined context.

## 1. Introduction

Concealed paintings, or early compositions that were later covered by other paintings, can provide a unique and illuminating insight into an artist’s creative process. They reveal unexpected details about the genesis of the work, offering insight into the artist’s techniques and inspirations [1,2,3]. In the last years, the non-invasive visualization of underpaintings and underdrawings in multi-layered paintings has been achieved through the complementary use of imaging and mapping techniques, such as reflectance imaging spectroscopy (RIS) [4,5] and macro X-ray fluorescence (MA-XRF) [6,7]. Several studies show that the integration of image data can significantly facilitate the interpretation and identification of internal materials related to the hidden iconographic features [8]. In 2015, the effectiveness of combining different imaging modalities led to the development of a multimodal registration and mosaicking algorithm that accurately aligns imaging data with reference color images acquired with high spatial sampling (300–500 pixels per inch) [9]. By registering X-radiographs, hyperspectral (HS), and RIS and XRF image cubes onto the reference images of the examined surface, several hidden features were revealed. The new algorithm was used to extract and combine information from both X-ray and near-infrared (NIR) images of a Vermeer painting, revealing the detail of a man’s face with a hat painted underneath the visible depiction. Similarly, Van der Snickt et al. applied X-ray radiography and hyper-spectral (HS) imaging in reflectance and transmission modes to reveal the underlying figurative composition in a painting by Magritte [10]. In the same years, Thurrowgood et al. presented a major revelation of a hidden portrait in a Degas painting by combining conventional IR reflectography, high-definition synchrotron radiation X-ray fluorescence microscopy (SR-XFM), and Raman X-ray scattering [11]. The XRF elemental maps were post-processed using a new methodology to gain a comprehensive technical understanding of the painting, which could not be obtained through individual conventional techniques.

In 2017, a hidden male portrait was revealed in the painting The Blue Room by Picasso by combining multispectral (MS) RIS, HS-RIS, and SR-XRF mapping [12].

Herens et al. applied three complementary non-invasive techniques—i.e., HS-RIS, MA-XRF, and Raman spectroscopy—to compositionally characterize the pigments of The Violin Player by Van Dongen and reveal the underlying female portrait painted by the same author [13].

In 2019, microanalysis combined with laboratory-scale MA-XRF and synchrotron MA-XRF allowed the visualization of a hidden landscape underlying the painting Exit from the Theater, calling into question its previous attribution to Honoré Daumier [14].

In the last few years, deep learning (DL) techniques have been introduced in painting studies to uncover concealed pictorial features. Specifically, a self-supervised method was proposed to separate the information contained in X-ray images of a painting by Goya, resulting in two hypothetical images that allowed for the visualization of the visible scene and the hidden subject, respectively [15]. The method was later updated to enable image separation without the need for labeled data. A composite loss function was introduced to guide the training of the separation network [16].

**Figure 1:**
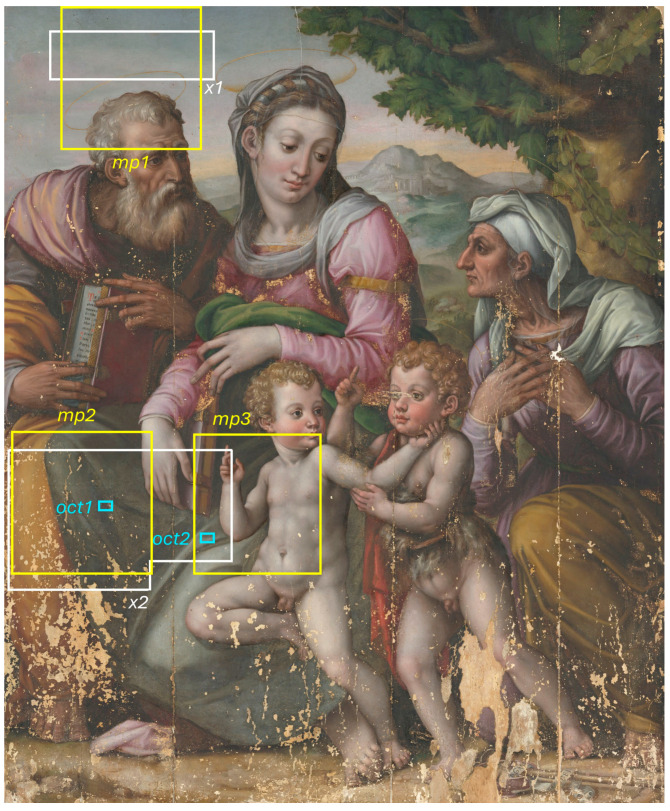
RGB image obtained from multispectral scanning, showing the regions analyzed with microprofilometry (*mp1*–*3*), macro X-ray fluorescence (*x1*,*2*), and optical coherence tomography (*oct1*,*2*).

This study analyses an oil painting (Figure 1) from the mid-16th century owned by the Uffizi Galleries. The painting was kept for decades in the museum storerooms of Palazzo Pitti until its recent transfer to the Opificio delle Pietre Dure, where it underwent restoration [17]. The results reported in this paper derive from the analyses carried out in the context of the conservation intervention. The painting, depicting the Holy Family, has recently been attributed to Domenico Beceri, a Florentine painter mentioned by Giorgio Vasari in his treatise, *Le Vite* [18]. In our previous work, we studied the stratigraphy, identified the pigments used by the artist, and mapped their distribution on the painted surface [19]. During the study of the materials, evidence emerged of paint layers underneath the visible scene of the Holy Family.

In this paper, we report the results of combined imaging and 3D analysis aimed at revealing the concealed depiction. A multimodal approach is used to visualize hidden features, highlight micrometric details, and reveal superimposed paint layers. Data are obtained through the joint application of MS-RIS, MA-XRF, microprofilometry (MP) [20], and spectral-domain optical coherent tomography (Sd-OCT) [21]. Multivariate statistical and classification methods are typically used to process the RIS image cube. Among others, principal component analysis (PCA) [22] reduces the data dimension, and spectral correlation mapping (SCM) [23] allows for the visualization of pigment distribution in the inner layers. The integration of the results provides the necessary information to achieve a comprehensive reconstruction of the hidden painting.

## 2. Materials and Methods

### 2.1. The Painting

The artwork under study is an oil-on-panel painting owned by the Uffizi Galleries. It dates back to the second half of the 16th century and depicts the Holy Family with St. John and St. Elizabeth. Recently, the painting has been attributed to Domenico Beceri, an artist who was active in Florence in the 16th century. Although little-known today, Beceri was recognized by Giorgio Vasari as a pupil of Domenico Puligo. Most of the observable damage of the painting was attributed to the events of World War II, rather than the 1966 flood in Florence, as previously believed, following the reconstruction of the work’s recent history.

### 2.2. Laser Scanning Microprofilometry (MP)

Microprofilometry is an incoherent-light interferometric technique allowing for distance measurements at the micrometric level [24]. The microprofilometer used is a custom-made device developed by the Heritage Science Group of CNR-INO (National Institute of Optics of the National Research Council) based on a conoscopic holography distance meter combined with a scanning system. The probe (ConoProbe1000 by Optimet, Jerusalem, Israel), comprising a laser diode at λ = 655 nm, a birefringent crystal placed between two polarizers, and a CCD camera, is mounted on two high-precision motorized linear stages for horizontal and vertical displacements.

For the present application, the probe was equipped with a 50 mm lens, which sets a stand-off distance of about 4 cm with 8 mm dynamic range. The resolution is 1 μm and 20 μm in the axial and lateral direction, respectively [25].

### 2.3. Macro X-ray Fluorescence (MA-XRF) Mapping

The MA-XRF scanner used in this work is a lightweight portable equipment developed in the framework of the Cultural Heritage Network of the Italian National Institute of Nuclear Physics, INFN-CHNet [26]. The instrument, which is described in detail in [27], comprises a measuring head mounted on three linear stages placed on top of a carbon fiber box containing the power supplies, the signal digitizer, and all the auxiliary elements. The measuring head is composed of an X-ray tube (Moxtek©, Orem, UT, USA, 40 kV maximum voltage, 0.1 mA maximum anode current, with Mo anode), a silicon drift detector (Amptek© XR100 SDD—Bedford, MA, USA, 50 mm^2^ effective active surface, 500 μm thickness), and a telemeter (Keyence IA-100), for keeping the sample-to-instrument distance constant when scanning. The motor stages Physik Instrumente©, Karlsruhe, Germany) allow the scan. The elemental distribution map is reconstructed by selecting an energy range that typically corresponds to the characteristic X-ray line of the element. The software assigns a greyscale level to each pixel based on the X-ray counts of the selected peak. White and black correspond to the maximum and minimum counts, respectively.

The experimental conditions for this campaign were: 40 kV anode voltage, 60 μA filament current, 10 mm/s scanning speed, 1 mm pixel size, beam diameter ~1 mm on the sample, with no helium flow.

### 2.4. Reflectance Imaging Spectroscopy (RIS)

The multispectral scanner used in this work was developed by the Heritage Science Group of CNR-INO. It combines filter-based pointwise spectral information in the range 395–2550 nm with whiskbroom scanning to acquire simultaneously 32 narrow-band images (16 Vis + 16 NIR) [28]. The lighting system comprises two low-voltage current-stabilized halogen lamps equipped with an aluminum back reflector (beam divergence ± 5°) and two narrow-spot high-power white LEDs (1 W, beam divergence ± 4.5°). A catoptric system (field of view, FOV, of 0.29°) focuses the light backscattered from the painting on the input end of a square-shaped fiber bundle, which delivers it to a set of Si and InGaAs photodiodes, each of them equipped with an interferential filter. The optical head, composed of the lighting system and the collecting optics, is placed in a 45°/0° illumination/detection geometry, following CIE indications for non-contact spectrophotometric measurements. It is moved by an XY scanning system with a 250 μm sampling step (4 points/mm) and 500 mm/s speed, resulting in a 3-h acquisition time for the maximum scanning area of 1 m^2^. A Z stage with a total run of 100 mm keeps the optical head in focus while scanning the surface, following the profile acquired by the autofocus system, i.e., a high-speed triangulation sensor. The instrument output is a set of perfectly superimposing monochromatic images, metrically correct and free from aberrations.

Five acquisitions were required to scan the entire painting, which were then stitched together into a single image cube. Proper calibration procedure was performed by measuring a certified standard reference (100% reflectance) as well as the background noise.

#### 2.4.1. Principal Component Analysis (PCA) and Spectral Correlation Mapping (SCM)

We analyzed RIS data cube with PCA [29], an unsupervised exploratory method widely used to reduce the dimensionality of RIS data without loss of information. It concentrates the spectral variance contained in the image cube into a smaller number of principal components (PCs) that still contains most of the information of the large dataset. The analysis selects the directions of maximum variance within the multivariate data space, which are orthogonal and thus uncorrelated. The information contained in the resulting PCs is never redundant [30].

Spectral mapping was performed with an automated classification method to directly compare two spectra through the criterion of similarity. The SCM algorithm considers the spectra of the image cube as vectors in an *n*-dimensional space, where *n* is the number of spectral bands. The angle between the reference (*r*) and the target spectra (*t*) measures their similarity, which is calculated with the Pearson’s Correlation Coefficient, *R* (Equation (1)):

(1) $$R=\sum_{i=1}^{n}\;\frac{{(t}_{i}-\bar{t})\cdot {(r}_{i}-\bar{r})}{{\left(\sum_{i=1}^{n}\;{{(t}_{i}-\bar{t})}^{2}\right)}^{1/2}\cdot {\left(\sum_{i=1}^{n}\;{{(r}_{i}-\bar{r})}^{2}\right)}^{1/2}}$$

where $\bar{t}$ and $\bar{r}$ are the mean values. SC maps are displayed as grey-scaled images where the pixel intensity is proportional to the angle between the vector representing the spectrum of each pixel and the reference (or endmember) being mapped. A small angle means a close match and a high-intensity value in the image plot. The correlation coefficient (*R*) ranges from −1 to 1, with 1 indicating maximum correlation. This method yields a more accurate classification than spectral angle mapping (SAM) [31], which does not account for negative correlation [32]. The SCM maps were generated using customized software developed in-house in Matlab^®^ (version 2021a). A spectral similarity range between 0.9 and 1 was chosen. Therefore, the areas highlighted on the maps indicate the presence of the reference pigment with high confidence.

#### 2.4.2. Reference Paint Mock-Ups

We used a series of oil paints on wooden support as a reference for SCM analysis. All samples were prepared by the Opificio delle Pietre Dure in 1996 according to late Medieval and Renaissance recipes. Pure powdered pigments (by Zecchi™, Florence, Italy), chemically characterized by FT-IR and SEM-EDS, were dispersed in stand oil (Zecchi™). The paint layers were applied on a preparatory background (gypsum and animal glue), previously finished with rabbit glue.

### 2.5. Spectral-Domain Optical Coherence Tomography (Sd-OCT)

Optical coherence tomography is a noninvasive interferometric method for the noncontact imaging of the internal microstructure of materials, which moderately scatter and/or absorb the probing light. OCT, originally developed for high-resolution cross-sectional analysis in biomedical studies, is well established in the cultural heritage field for the thickness measurement of varnishes and semi-transparent paints, sometimes allowing the visualization of underdrawings [33,34].

The OCT device used in this study is a Thorlabs Telesto-II that works at 1300 nm with 100 nm bandwidth, entailing an axial resolution of 5.5 µm in air. The lens (with effective focal length, EFL = 36 mm) sets a lateral resolution of 13 µm and a maximum field of view (FOV) of 10 × 10 mm^2^, with a 3.5 mm imaging depth. The depth information is acquired using a Fast Fourier Transformation (FFT). The system is controlled via 64-bit software running on a high-performance computer. The 3D scanning probe with an integrated video camera allows for high-speed imaging (76 kHz) for rapid volume acquisition and live display. The 3D and 2D tomograms were acquired with a voxel size of 6.5 × 6.5 × 3.5 µm^3^ and a pixel size of 3.5 µm^2^, respectively.

## 3. Results

Initial visual observations of the painting under raking light revealed morphological features that can be traced back to paint brushstrokes underneath the visible depiction of the Holy Family. These latter details were particularly evident in specific areas of the painting, namely the sky above St. Joseph’s head, the greenish dress of the Virgin, and the body of the infant Jesus. The areas highlighted in Figure 1 were measured using microprofilometry (*mp1*–*3*), followed by macro X-ray fluorescence (*x1*–*2*) and optical coherence tomography (*oct1*,*2*). The entire surface of the painting was scanned using multispectral reflectography and the image data from the different techniques were cross-referenced to guide the reconstruction of the concealed painting.

### 3.1. Surface Micromorphology

**Figure 2:**
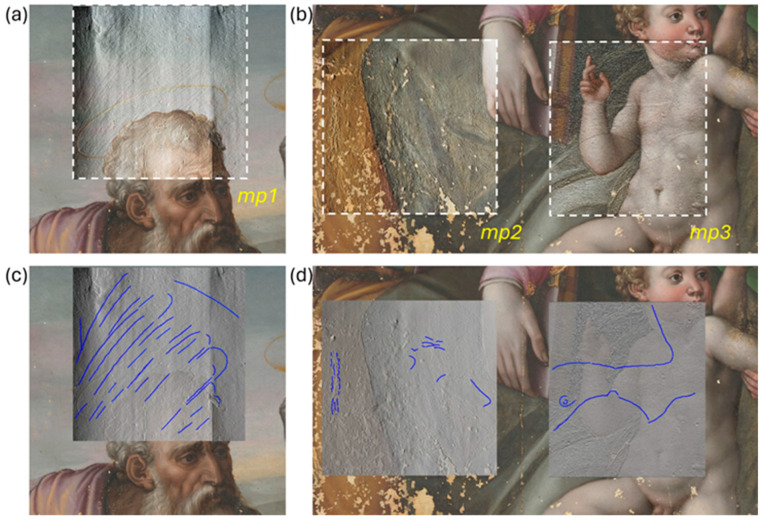
Microprofilometry results: topographic maps of *mp1* (**a**) and *mp2*,*3* (**b**) overlaid with 50% transparency on the RGB image to enhance the 3-dimensional micrometric features, which are highlighted by the blue line on the same maps, displayed as raking light images (**c**,**d**).

From MP measurements, we generated an elevation dataset of three areas on the painting surface, enabling the detection of micrometric details related to the covered depiction. The resulting topographic maps were processed and rendered as raking light images in Adobe Photoshop^®^ (version CC 2023) and the direction of the virtual light source was optimized for best visualization of the micrometric features. These latter were then registered on the RGB image using the overlay option with 50% transparency for the raking light image (Figure 2a,b).

From the MP topographic maps, acquired with a 50 µm sampling step, we identified the direction of the brushstrokes, which are marked with a blue line in Figure 2c,d. In the *mp1* region, the surface morphology resembles that of a bird’s wing extending from the sky area to below St. Joseph’s head. Area *mp3* displays curved lines that may resemble a woman’s breast and an eye, and area *mp2* shows limited details that could replicate the physiognomic features of a face.

To correlate the wooden panel morphology to the RGB image, we overlayed the 3D low-resolution (250 µm pixel size) model, registered during the multispectral acquisition as the autofocus output, with the color image (Figure S1 in the Supplementary Materials). This 3D and color data set is self-co-registered: its overlay gives an overall view of the painting where the elevation information is perfectly superimposed with the spectrophotometric one for each pixel.

### 3.2. Underdrawings

**Figure 3:**
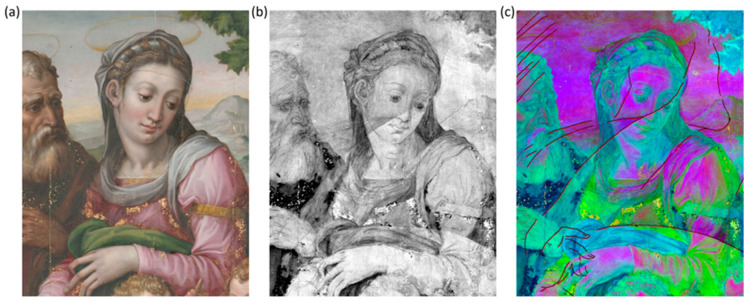
Detail of the visible depiction in the RGB image (**a**) and of its underdrawing in the NIR at 1830 nm (**b**); PC color-composite image (**c**) obtained by combining PC1, 2, and 3 (inverted).

**Figure 4:**
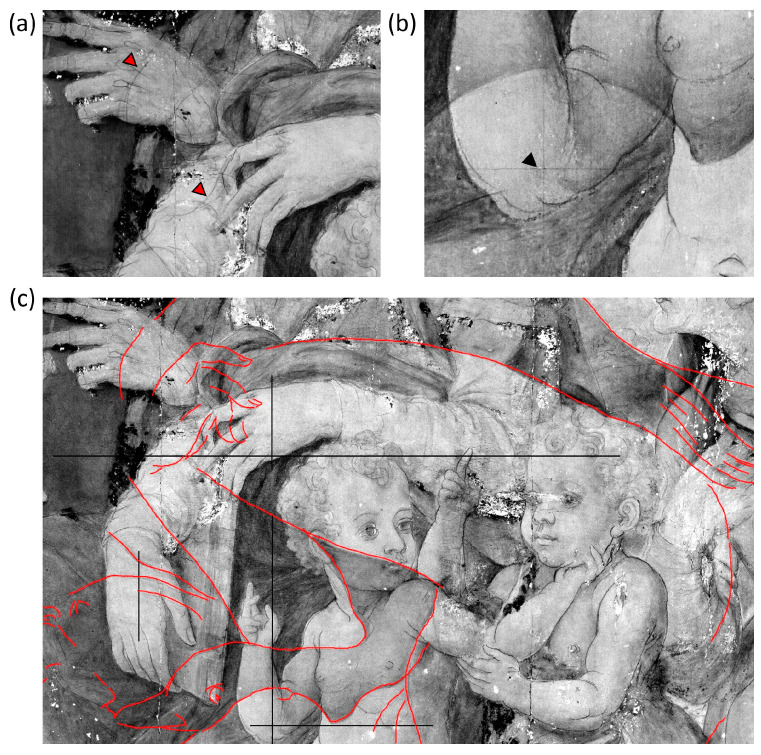
Details of the underdrawing of the concealed painting indicated by the red markers in the image at 2100 nm (**a**); detail of the grid lines pointed out by the black marker in the image at 1830 nm (**b**); (**c**) reconstruction of the concealed underdrawing (red line) and grid segments (black lines) in the image at 2100 nm.

Reflectance imaging in the NIR range enabled the visualization of distinct graphic lines beneath the superficial pictorial layers that are ascribable to the underdrawing of the Holy Family. A representative detail is reported in Figure 3. The sketch that outlines the figures is visible from around 1200 nm and becomes particularly evident at 1830 nm (Figure 3b). The stroke reveals a confident and decisive approach, which can be derived from Michelangelo’s lesson. We applied principal component analysis to the image cube, both to highlight differences in the spectral response of the painting surface and to increase the interpretability of features underlying the visible paint layer. Figure 3c displays the composite image obtained by combining PC1, 2, and 3 (inverted) in the trichromatic RGB space, which accounts for 87.4%, 7.7%, and 2.1% of the total variability, respectively. The regions with different spectral behavior are outlined by the red lines, which may be attributed to the presence of an underpainting. In the NIR images from 2100 nm onwards, additional light and subtle graphic features become visible that can be attributed to a second underlying drawing (Figure 4a). Poorly defined details, presumably watercolored, and some evidence of pentimenti were also observed. The visibility of the strokes varies depending on the area due to the absorption or diffusion properties of the overlying paints in the NIR. Previous examination of the painting stratigraphy revealed a 5–10 μm-thick paint layer made of a mixture of lead white and carbon black [19]. This layer is probably an *imprimitura* applied over the underlying painting to create an opaque background for the subsequent painting. It is possible that the priming layer was thickened in certain areas to increase opacity, which would further obscure the underdrawing. The absorption properties of both sketches suggest the use of a carbon-based material. The consistency and variability of the marks are compatible with the use of a smooth yet brittle material, such as black charcoal.

Upon careful analysis of the NIR image at 1830 nm, we observed a series of orthogonal lines where the overpainting is transparent enough to allow the penetration of the incident radiation (Figure 4b). These lines can be traced back to a grid, which is marked in black in Figure 4c, along with the underdrawing (dark red line) of the concealed painting. Some lacunae in the painting show fragments of the grid that appear to have been drawn directly onto the preparatory layer, suggesting the use of the grid for the underdrawing of the concealed painting rather than for the Holy Family. This technique was commonly used to transpose and resize graphical subjects, as seen in other well-known paintings of the same period [35]. Figure S2 in the Supplementary Materials shows the image at 1830 nm along with the complete reconstruction of the grid and underdrawing based on RIS analysis.

### 3.3. Underpainting

**Figure 5:**
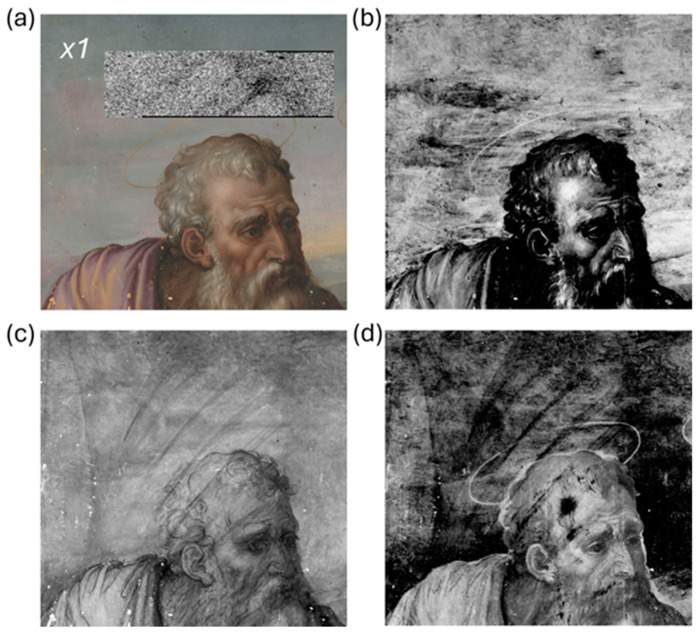
MA-XRF and RIS results: Pb XRF map (area *x1*) overlaid on the RGB image (**a**); SC map of lead white (**b**); NIR image at 2550 nm (**c**); SC map of yellow ochre (**d**).

MA-XRF and SC maps obtained with all the pigments identified in the previous study were examined and compared to highlight the hidden details of the underdrawing and to extract compositional information on the inner layers. The XRF map in Figure 5a shows the contour of the bird’s wing above St. Joseph’s head (area *x1*) and suggests the use of lead white (2 PbCO_3_·Pb(OH)_2_) for this detail. The SC map obtained using lead white as an external endmember (Figure 5b) shows high-intensity pixels in the sky area, indicating the use of this pigment for the Holy Family, which is consistent with the mixture previously identified in this region, composed of smalt, lead white, and carbon black [19]. The detection capability of the multispectral analysis is limited in this case due to the presence of the pigment in the surface layer, which overlaps any signal from the innermost layers. In contrast, the NIR image at 2550 nm (Figure 5c) clearly shows the wing contours and the feathers under St. Joseph’s head. The SC map of yellow ochre (Figure 5d) suggests its use in the covert painting for the wing, as well as St Joseph’s skin and hair in the visible painting.

**Figure 6:**
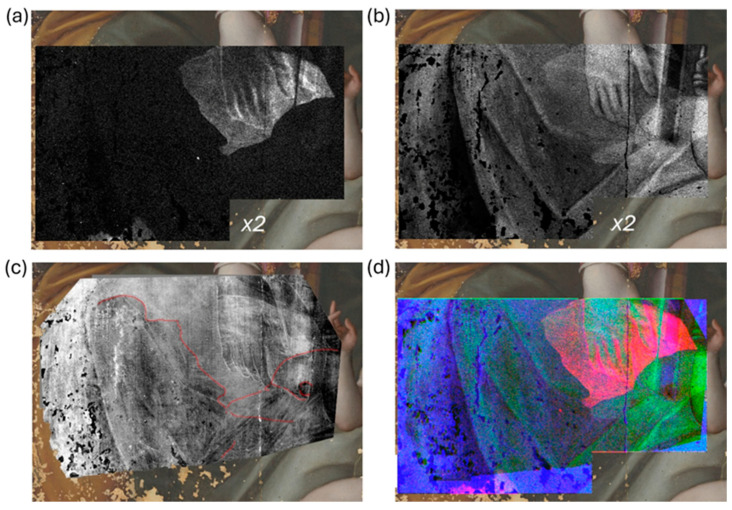
Detail of the Virgin’s dress (area *x2*): Cu XRF map (**a**); Pb XRF map (**b**); PC3 inverted image with details outlined in red; color-composite image (**d**) obtained by combining (**a**–**c**).

The XRF analysis on the Virgin’s dress (area *x2*) showed the presence of Cu (Figure 6a) and Pb (Figure 6b). The distribution of Cu reveals a well-defined shape under the Virgin’s hand, while the distribution of Pb allows for the clear visualization of a bird’s head. The eye of the bird can be perfectly superimposed on the morphological feature already observed in the MP map (*mp3* in Figure 2d). The information of PC3 (Figure 6c) was enhanced by adjusting the intensity levels to increase the overall contrast of the region of interest and by inverting the brightness values. This allowed highlighting the presence of a darker region revealing a face-like profile on the left of the bird’s beak, whose contours are outlined in red. The color-composite image in Figure 6d is obtained by combining the Cu map, the Pb map, and the PC3 image as red, green, and blue channels, enhancing the visualization of the concealed painting.

### 3.4. Virtual Reconstruction

**Figure 7:**
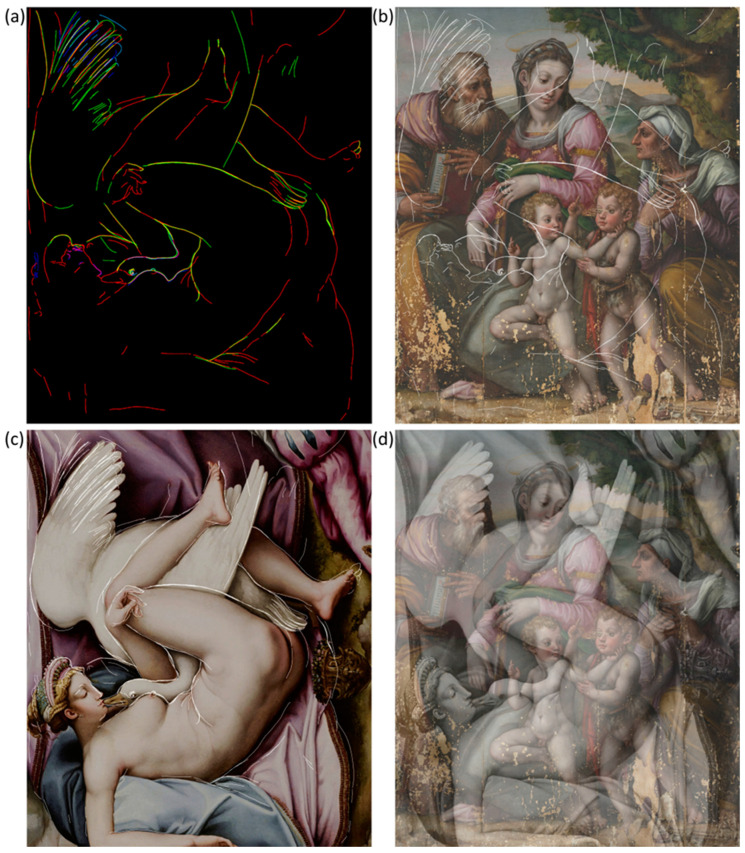
Virtual reconstruction of the concealed depiction: (**a**) combined RGB profile, including the information obtained from RIS (red line), SCM (green line), and MP (blue line); (**b**) single line profile overlaid on the RGB image of the Holy Family; (**c**) virtual drawing superimposed over the Leda and the Swan painting belonging to the Accademia di Bergamo (©Fondazione Accademia Carrara, Bergamo); (**d**) Leda painting overlaid on the RGB image of the Holy Family with 50% transparency.

The concealed depiction was virtually reconstructed (Figure 7a) by integrating all the information extracted from the results of microprofilometry and multispectral reflectance imaging. Details observed in the NIR and PC images are outlined in red, features evidenced in SC maps are delineated in green, and the micrometric morphology highlighted by MP is contoured in blue. The combined RGB profile image is created by merging the former digital drawings previously attributed to the red, green, and blue channels in Photoshop by linear combination. Hence, when two or three drawings overlap, the resulting hue is magenta (red + blue), yellow (red + green), cyan (green + blue), and white (red + green + blue). The final drawing is shown as a single white line overlaid on the RGB image of the Holy Family in Figure 7b, allowing for a clear visualization of the concealed depiction. The virtual reconstruction portrays a female figure embracing a swan with outstretched wings, representing the myth of Leda and the Swan, as originally depicted by Michelangelo in a lost painting. This subject has been reproduced in numerous replicas in later years (see the Discussion section). The scene resembles the homonymous painting in the Accademia Carrara in Bergamo, more so than any other known version. Figure 7c, showing the virtual drawing superimposed over Leda and the Swan, demonstrates this close match. The painting is then overlaid with 50% transparency on the RGB image of the Holy Family to reproduce the hypothetical original depiction beneath the visible one (Figure 7d).

**Figure 8:**
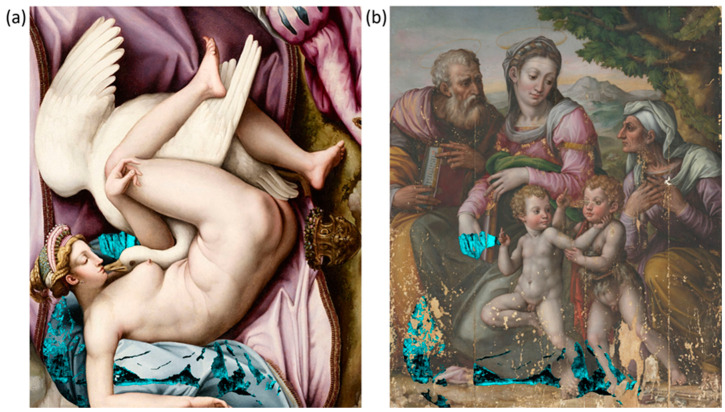
SCM of azurite displayed in light blue and overlaid on the Leda painting, ©Fondazione Accademia Carrara, Bergamo (**a**), and on the Holy Family painting (**b**).

Given the detection of Cu in the area near the swan’s head (Figure 6a), spectral correlation maps were produced by referencing Cu-based pigments, namely azurite, malachite, copper green, and verdigris. A high spectral similarity was found with azurite (2CuCO_3_-Cu(OH)_2_) in the area near the swan’s head and throughout the region corresponding to the blue drapery on which Leda lies. The distribution of azurite is shown in light blue superimposed on the Leda painting (Figure 8a) and the RGB image of the Holy Family (Figure 8b). SCM can only detect azurite in areas where the overpaint is transparent to incident radiation. Therefore, the contribution of azurite from the inner layers is largely obscured by the Virgin’s robe and the ground, but it can be seen under the yellowish robe of St Joseph and the legs of the children.

SCM provided additional information on the pigments used for the swan feathers. The external endmember ochre yellow and carbon black exhibited a high spectral correlation with the wing on the left (Figure 5d and Figure S3) and the tail below Leda’s left leg (Figure S3). The detection of Pb by MA-XRF in correspondence of the upper wing and the swan’s head (Figure 5a and Figure 6b) suggests that lead white was used in a mixture along with yellow ochre and carbon black.

### 3.5. Is Leda’s Face Painted?

**Figure 9:**
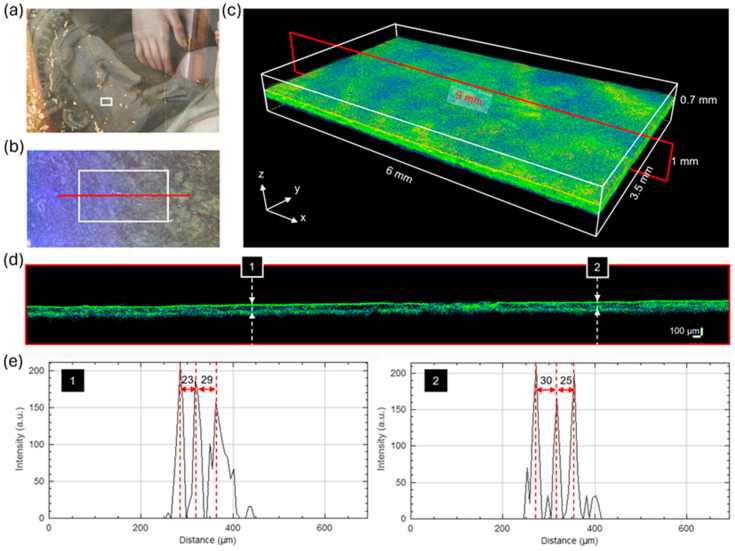
OCT analysis on area *oct1*: (**a**) detail of the painting with the Leda painting overlaid on the RGB image; (**b**) micro showing the examined area (white rectangle) and the position of the XZ section (red line); (**c**) OCT tomo-cube with the XZ section highlighted by the red rectangle; (**d**) XZ section with vertical dashed lines (1 and 2) indicating the position of the Z profiles; (**e**) signal intensity plotted as a function of depth, with the three main peaks (red dashed lines) corresponding to the air/paint, paint/underpaint, and underpaint/preparation interfaces.

The results of the imaging techniques have disclosed Leda and the Swan beneath the visible depiction of the Holy Family. Albeit the underdrawing is detailed, it is uncertain whether the underlying painting was accomplished before being covered with the Holy Family. The visibility of the inner layers is limited underneath the Virgin’s robe, due to the high optical opacity of the pigment mixture, making it difficult to determine the degree of finishing of Leda’s face. The detail evidenced by the inverted PC3 (Figure 6c) suggests the presence of a uniformly applied material in the area where Leda’s face should have been painted. To investigate the stratigraphy non-invasively, we carried out OCT measurements. The results on areas *oct1* and *oct2* are reported in Figure 9 and Figure S3, respectively. We acquired the 3D tomogram on area *oct1* (size: 6 × 3.5 × 0.7 mm^3^) highlighted in white in Figure 9a,b. The red line in the magnified image (Figure 9b) indicates the position of the XZ section (size: 9 × 0.7 mm^2^). The pseudo-color rendering of the tomo-cube showing the micro-morphology of the surface is shown in Figure 9c. The XZ section is reported in Figure 9d, with the vertical dashed lines (1 and 2) indicating the position where the intensity profiles (Figure 9e) along the *z*-axis were extracted. Both plots show three main peaks (red dashed lines): the first corresponds to the air/paint interface, and the second and the third are attributed to the paint/underpaint and underpaint/preparation interface, respectively. The second peak indicates the presence of an additional paint layer, which may correspond to the concealed painting. We used *n* = 1.5 for the refractive index to correct the optical distance between the interfaces. Therefore, the resultant thickness range was 23–30 µm for the outermost layer of paint (the Virgin’s robe) and 25–29 µm for the inner layer of paint (possibly Leda’s flesh tone).

## 4. Discussion

The unveiled depiction represents the mythical union of Leda, Queen of Sparta, with the god Jupiter in the form of a swan. This subject appears to have been inspired by a depiction created by Michelangelo for Alfonso d’Este, Duke of Ferrara, in 1530 [36]. For reasons that remain unclear, the artist never delivered the painting to the duke, instead sending it, and his cartoon, to France with his assistant, Antonio Mini. Scholars agree that Michelangelo’s original painting is now irretrievably lost. It is said to have been destroyed in the 17th century by order of Queen Anne of Austria, who objected to its ‘lasciviousness’ [37]. The fate of the cartoon, however, remains an open question. According to Vasari, the cartoon came into the possession of the collector Bernardo Vecchietti, after being brought back to Florence [38]. In 1691, a drawing by Michelangelo depicting Leda and the Swan was recorded in an inventory of the French Royal Collection. The drawing was to be destroyed in an act of iconoclasm, as it is suggested by its absence from later inventories [39]. The Royal Academy of Arts in London currently holds a cartoon that some believe to be the one mentioned by Vasari, or at least a direct copy of the original [27]. Others, however, attribute the drawing to Rosso Fiorentino [40].

According to Vasari [18] and Condivi [41], in Michelangelo’s original painting, the unhatched eggs with the Dioscures (Castor and Pollux), born of the sacred union between Leda and Jupiter, were also depicted on the periphery of the main scene. However, it is assumed that the original cartoon did not contain this figurative element, as many surviving reproductions do not show it, replacing it with an ornamental drapery in the right-hand corner. Traces of the drapery have also been detected in the painting under examination, consistently with the original cartoon. Of all the known reproductions, the virtual reconstruction bears a striking resemblance to Leda and the Swan in the Accademia Carrara in Bergamo. Another similar version is located at the Museo Civico Correr in Venice, attributed to an anonymous Venetian painter of the late 16th century. The authorship of the painting in Bergamo is still uncertain, but some scholars attribute it to Francesco Ubertini, also known as Bachiacca (1494–1557). He may have been a student of Perugino in Florence and later influenced by Andrea del Sarto, as well as Pontormo and Vasari. He also created several cartoons for tapestries [42]. Bachiacca’s painting is actually smaller (57 × 78 cm) and mirrored compared to the unveiled painting, but the depictions seem uncanny, both in terms of proportions and profile lines as well as colors in the painted areas. The fact that this is a transposed and rescaled copy is also suggested by the presence of the grid lines. Considering the close contemporaneity of the cited paintings with the one under study, it is possible that they are all reproductions of the same cartoon, the original Leda and the Swan by Michelangelo [17].

The cross-analysis of the imaging data provided by the different techniques was based on a meticulous time-consuming comparison that allowed all the information to be gathered in a manual graphical reconstruction of the hidden depiction. The use of an automated approach based on deep-learning methods—e.g., convolutional neural network (CNN) [43]—for the recognition of details attributable to underdrawing could be a promising way to reduce the analysis time in studies of this type. However, the presence of more than one underdrawing, as in our case, would increase the level of complexity, requiring a system capable of distinguishing overlapping graphic features belonging to different sketches. On the other hand, the presence of pictorial layers below the visible surface can be effectively detected and mapped by multivariate analysis of reflectance imaging data. To overcome the visibility limitation given by optically opaque overpainting to incident NIR radiation, the combination of complementary techniques with different detection capabilities, such as the macro-XRF applied in this study, is the most promising avenue to date.

## 5. Conclusions

We have non-invasively unveiled the hidden depiction of Leda and the Swan covered by the visible scene of the Holy Family. The reconstruction was achieved through the combined application of microprofilometry, reflectance imaging spectroscopy, X-ray fluorescence, and optical coherence tomography. The 3D survey allowed for the detection of micrometric features, such as the brushstrokes of the hidden painting, which were related to the underdrawing discovered in the NIR images. The principal component analysis on the image cube enabled evidence of the diverse spectral behavior of the inner materials, suggesting the presence of painted areas underneath the outermost paint layer. Specifically, PCA revealed the presence of a uniformly applied material in the area where Leda’s face should have been painted. This finding is consistent with the pictorial interfaces observed in the stratigraphy by OCT. The cross-referenced analysis of X-ray fluorescence and spectral correlation maps allowed for the identification of the main pigments used for the drapery (azurite) and for the swan’s feathers (a mixture of lead white, yellow ochre, and carbon black). The underlying painted subject, Leda and the Swan, is clearly inspired by a long-lost work by Michelangelo and its replicates. This information provides relevant support for situating Beceri and his production within a more defined context.

## Data Availability

The data presented in this study are available on request from the corresponding author.

## Supplementary Materials

The following supporting information can be downloaded at: https://www.mdpi.com/article/10.3390/jimaging10080175/s1, Figure S1. Topographic map acquired by the autofocus device during multispectral scanning overlaid on the RGB image; Figure S2. NIR image at 1830 nm with the complete reconstruction of the grid (black) and underdrawing (red) based on RIS analysis; Figure S3. SCM of yellow ochre (yellow) and carbon black (green) overlaid on the Leda and Swan, ©Fondazione Accademia Carrara, Bergamo (a), and on the Holy Family (b).; Figure S4. OCT analysis on area oct2: detail of the RGB image (a) and Leda’s painting (b) showing the area (white square) where the tomo-cube was acquired; macro (c) with red lines indicating the position of the extracted XZ section (red line); XZ sections with the distance between air/paint and paint/preparation interface reported in yellow (d).
