# Supplementary material for: Disclosure of a Concealed Michelangelo-Inspired Depiction in a 16th-Century Painting
Source: J Imaging. 2024 Jul 23;10(8):175. doi: 10.3390/jimaging10080175 (PMC11355436; doi:10.3390/jimaging10080175)
Supplement: Supplementary file 1 [file jimaging-10-00175-s001.zip › jimaging-3113808-supplementary.pdf]

Disclosure of a concealed Michelangelo-inspired depiction in a 16th-century painting

Alice Dal Fovo [1,*], Margherita Morello [2], Anna Mazzinghi [3,4], Caterina Toso [5], Enrico Pampaloni [1], Raffaella Fontana [1]

1 National Research Council – National Institute of Optics (CNR-INO), Largo E. Fermi 6, 50125, Florence, Italy
2 OPD-Scuola di Alta Formazione e Studio, Via Alfani 78, 50121 Florence, Italy
3 University of Florence, Department of Physics and Astronomy, Via Sansone 1, 50019, Sesto Fiorentino, Italy
4 National Institute of Nuclear Physics (INFN), Florence division, Via Bruno Rossi, 1, 50019, Sesto Fiorentino, Italy
5 Opificio delle Pietre Dure, V.le Filippo Strozzi, 1, 50129, Florence, Italy
* Correspondence: alice.dalfovo@ino.cnr.it

Supplementary material

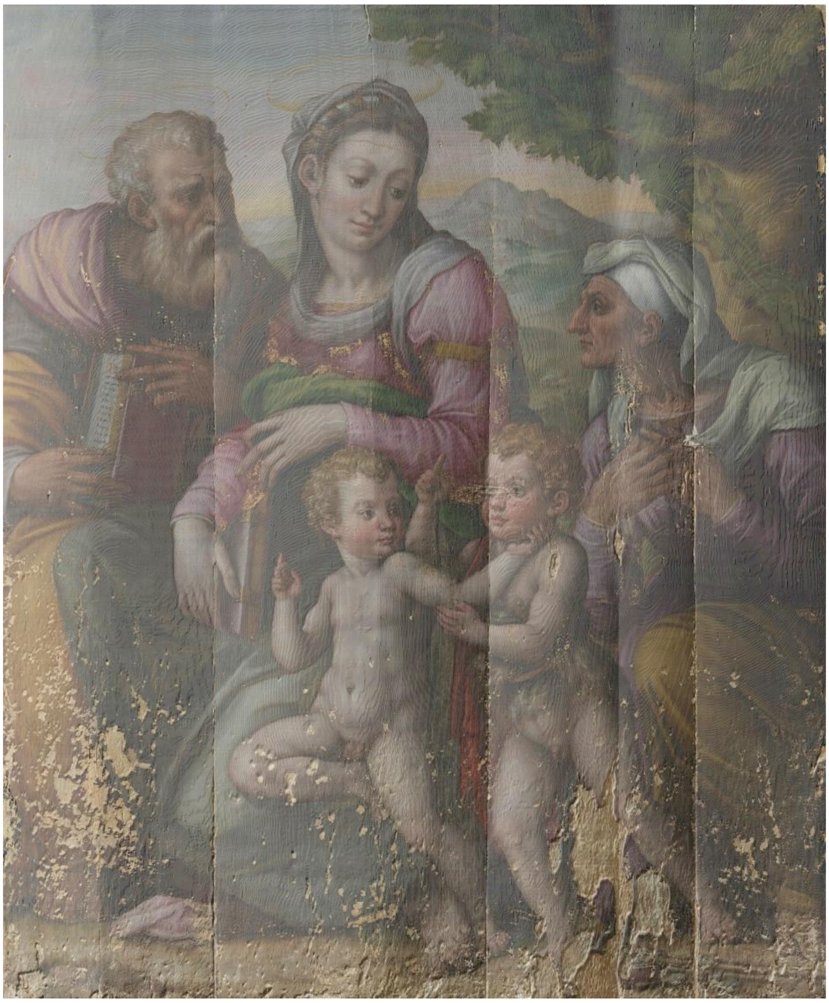

Figure S1. Topographic map acquired by the autofocus device during multispectral scanning overlaid on the RGB image.

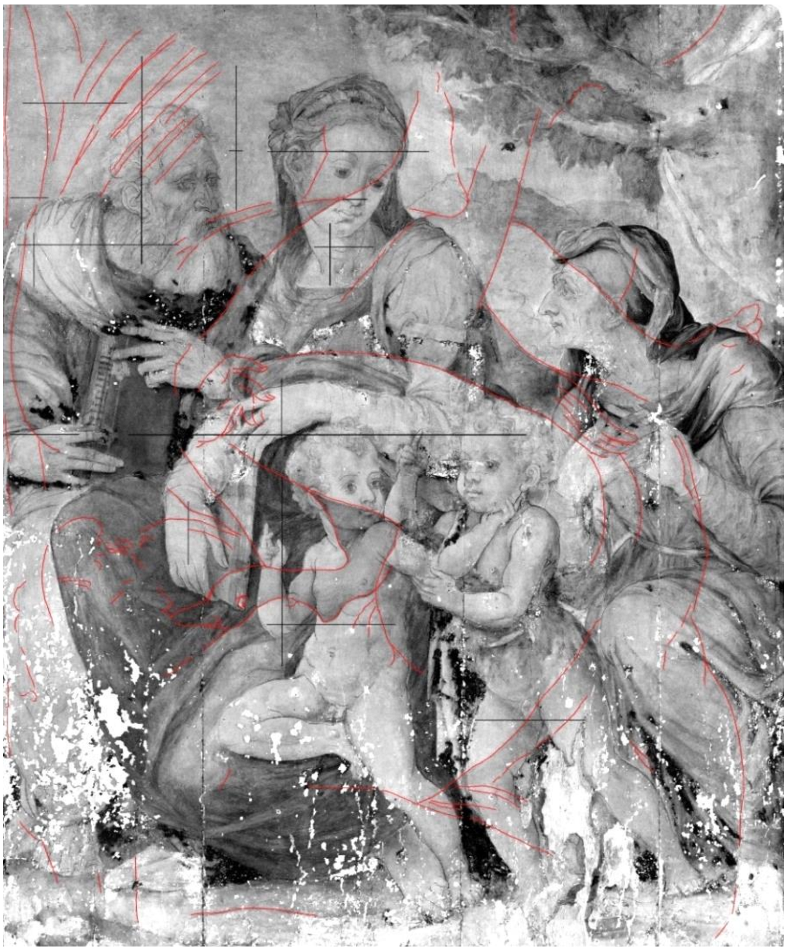

Figure S2. NIR image at 1830 nm with the complete reconstruction of the grid (black) and underdrawing (red) based on RIS analysis.

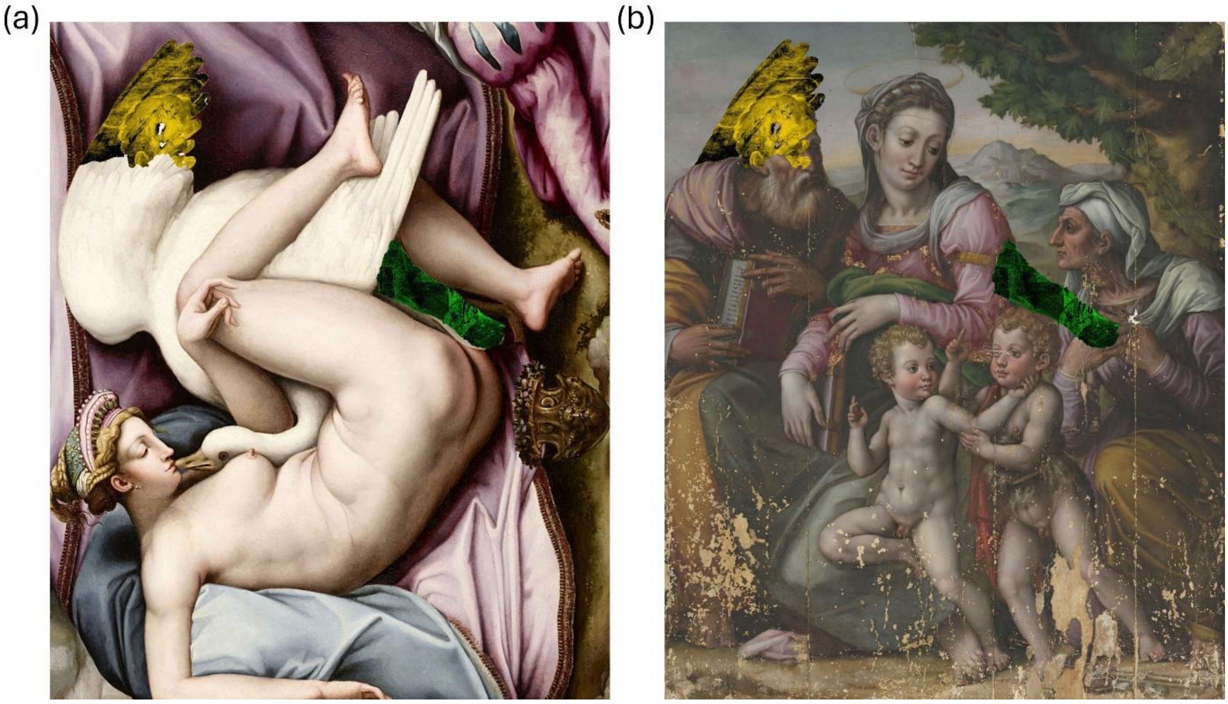

Figure S3. SCM of yellow ochre (yellow) and carbon black (green) overlaid on the Leda and Swan, ©Fondazione Accademia Carrara, Bergamo (a), and on the Holy Family (b).

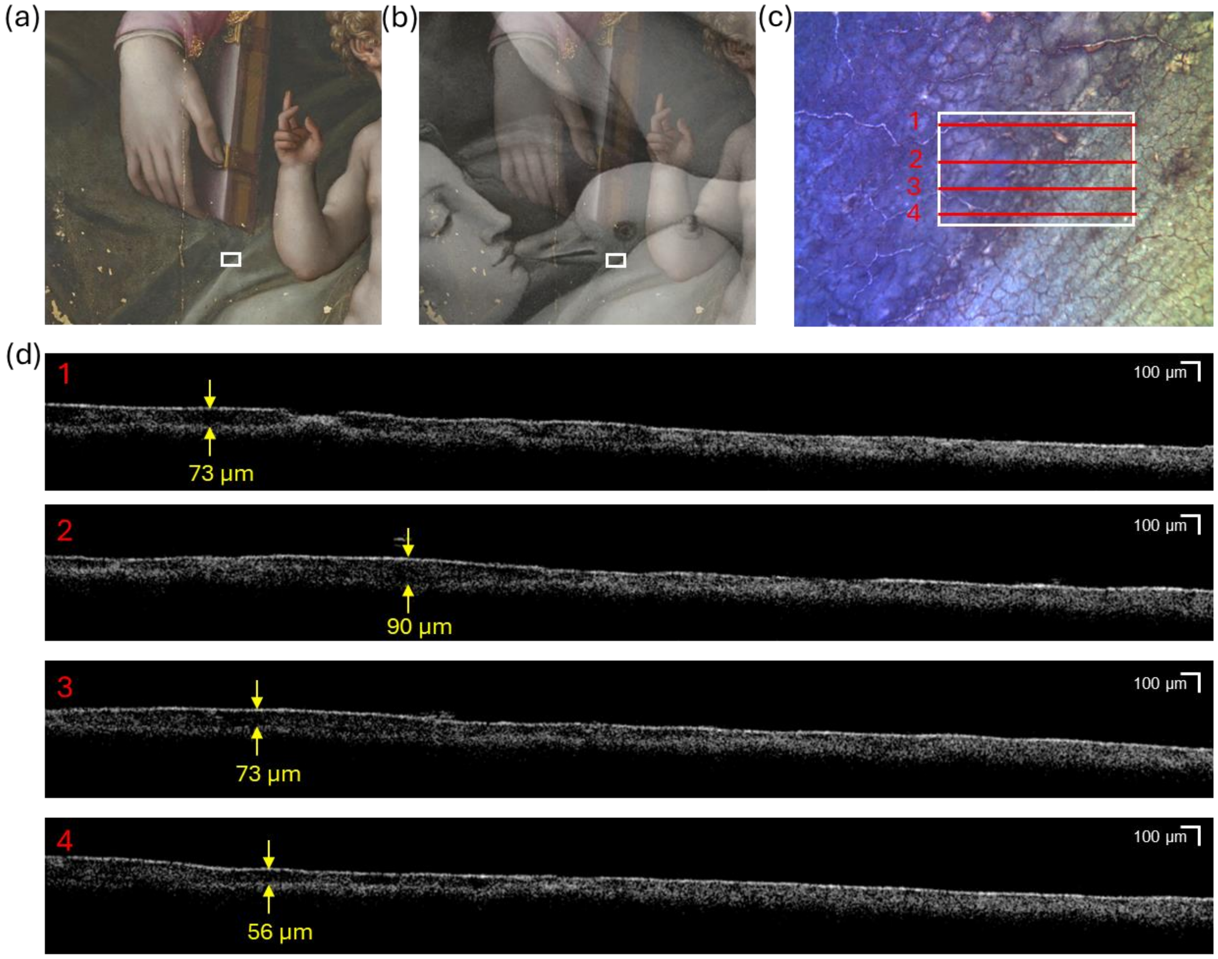

Figure S4. OCT analysis on area oct2: detail of the RGB image (a) and Leda's painting (b) showing the area (white square) where the tomo-cube was acquired; macro (c) with red lines indicating the position of the extracted XZ section (red line); XZ sections with the distance between air/paint and paint/preparation interface reported in yellow (d).
